# *QuickStats:* Percentage* of Adults^†^ Who Were in Families Having Problems Paying Medical Bills During the Previous 12 Months,^§^ by Race and Selected Hispanic^¶^ Origin Subgroups — National Health Interview Survey, United States, 2020−2021**

**DOI:** 10.15585/mmwr.mm7215a8

**Published:** 2023-04-14

**Figure:**
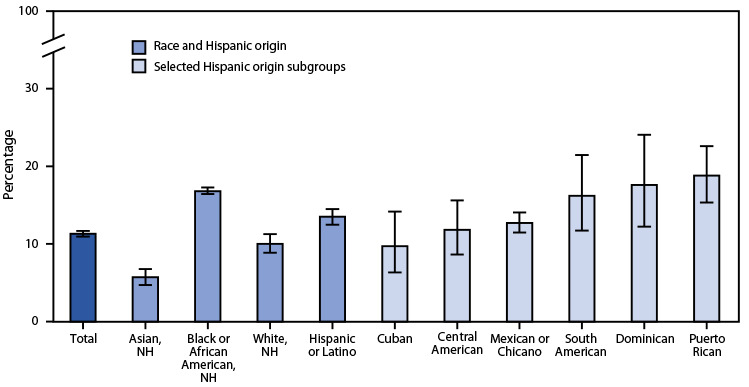
During 2020–2021, the percentage of U.S. adults who were in families having problems paying medical bills during the previous 12 months was 11.3%. Non-Hispanic Black or African American adults (16.8%) were most likely to be in families having problems paying medical bills followed by Hispanic or Latino (13.5%), non-Hispanic White (10.0%), and non-Hispanic Asian (5.7%) adults. Among the Hispanic or Latino origin subgroups shown, the percentage of adults in families having problems paying medical bills ranged from 9.7% among Cuban to 18.8% among Puerto Rican adults.

**Source:** National Health Interview Survey, 2020 and 2021 data. https://www.cdc.gov/nchs/nhis.htm

