# *Erratum:* Vol. 72, No. 15

**DOI:** 10.15585/mmwr.mm7223a7

**Published:** 2023-06-09

In the report, “*QuickStats*: Percentage of Adults Who Were in Families Having Problems Paying Medical Bills During the Previous 12 Months, by Race and Selected Hispanic Origin Subgroups — National Health Interview Survey, United States, 2020−2021,” on page 414, in the graph, the error bars for the Black or African American, NH category should have denoted a 95% CI of **1.1–1.3** and for the White, NH category should have denoted a 95% CI of **0.4–0.5**.

